# Two Novel Hydroxymethylbilane Synthase Splicing Mutations Predispose to Acute Intermittent Porphyria

**DOI:** 10.3390/ijms222011008

**Published:** 2021-10-12

**Authors:** Yanping Zhang, Han Xiao, Qiuhong Xiong, Changxin Wu, Ping Li

**Affiliations:** Key Laboratory of Chemical Biology and Molecular Engineering of National Ministry of Education, Institutes of Biomedical Sciences, Shanxi University, Taiyuan 030006, China; 201923105006@email.sxu.edu.cn (Y.Z.); hanxiao@sxu.edu.cn (H.X.); qxiong@sxu.edu.cn (Q.X.)

**Keywords:** acute intermittent porphyria, hydroxymethylbilane synthase, HMBS, splicing mutation, novel mutation

## Abstract

Acute intermittent porphyria (AIP) is an autosomal dominant genetic disease caused by a lack or decrease in hydroxymethylbilane synthase (HMBS) activity. It is characterized by acute nerve and visceral attacks caused by factors in the process of heme synthesis. The penetrance rate of this disease is low, and the heterogeneity is strong. Here, we reported two novel *HMBS* mutations from two unrelated Chinese AIP patients and confirmed the pathogenicity of these two mutations. We found the *HMBS* c.760–771+2delCTGAGGCACCTGGTinsGCTGCATCGCTGAA and *HMBS* c.88-1G>C mutations by second-generation sequencing and Sanger sequencing. The in vitro expression analysis showed that these mutations caused abnormal *HMBS* mRNA splicing and premature termination or partial missing of HMBS protein. Homologous modeling analysis showed that the HMBS mutants lacked the amino acids which are crucial for the enzyme activity or the protein stability. Consistently, enzyme activity analysis confirmed that the HMBS mutants’ overexpression cells exhibited the reduced enzyme activity compared with the HMBS wildtype overexpression cells. Our study identified and confirmed two novel pathogenic *HMBS* mutations which will expand the molecular heterogeneity of AIP and provide further scientific basis for the clinical diagnosis of AIP.

## 1. Introduction

Porphyria is a group of metabolic diseases caused by the abnormal enzyme activity during the heme synthesis pathway [1,2]. In general, porphyria can be divided into hepatic porphyria and erythropoietic porphyria according to different sites of heme precursor accumulation [3]. Acute intermittent porphyria (AIP; OMIM#176000) is a type of hepatic porphyria caused by abnormal hydroxymethylbilane synthase (HMBS; EC 2.5.1.61), also known as porphyrinogen deaminase (PBGD) [4]. AIP has the characteristics of low penetrance [5]. Population-based research showed that the acute exacerbation penetrance rate of mutation carriers is as low as 1%. However, acute attacks in individuals are often life-threatening and are usually accompanied by repeated acute attacks of neurological and visceral manifestations, including abdominal pain, vomiting, constipation, hypertension, tachycardia, peripheral neuropathy, and mental disorders [6]. Acute attacks of AIP are caused by a variety of factors, including certain drugs, fasting, and hormone changes. All of these factors induce 5′-aminolevulinic acid synthase 1 (ALAS 1; EC 2.3.1.37), the first rate-limiting enzyme of the heme biosynthetic pathway. When liver ALAS1 is induced, a large amount of intermediate product is produced, HMBS enzyme activity is insufficient, neurotoxic porphyrin precursor 5′-aminolevulinic acid (ALA) and porphyrinogen (PBG) accumulate in the liver, and they are secreted into the plasma and out through urine [7,8,9,10,11]. Therefore, high levels of ALA and PBG in urine are often used clinically as typical biochemical indicators of AIP. In theory, the inheritance of *HMBS* mutations is equal between men and women [12,13]. However, the vast majority of patients with clinical manifestations of porphyria are women, and they usually develop during or after puberty [13,14]. This is usually due to hormonal changes in the luteal phase of the menstrual cycle, combined with other predisposing factors to trigger the attack [15].

The human *HMBS* gene (NCBI Reference Sequence: NC-000011.10) coding sequences are spread over 15 exons, and which has been confirmed to produce two types of transcripts: erythroid isoform (containing exons 2–15) and housekeeping isoform (containing exon 1 and exons 3–15). The defected housekeeping form (NCBI reference sequence NM_000190.4) often leads to reduced enzyme activity causing AIP [16,17]. The crystal structure of human HMBS housekeeping form shows that it is composed of three different α/β domains of similar size, and the active site is located between domains 1 and 2 [18,19].

So far, more than 500 *HMBS* gene mutations that cause AIP have been reported in the Human Gene Mutation Database (HGMD) [20], of which there are 108 mutations related to site splicing. Here, we identified two novel mutations in the *HMBS* gene related to splicing: c.760–771+2delCTGAGGCACCTGGTinsGCTGCATCGCTGAA and c.88-1G>C. These two mutations change the splicing site that is essential for splicing. The purpose of this study is to describe the molecular effects and mechanisms of these novel *HMBS* mutations causing AIP.

## 2. Results

### 2.1. Identification of Novel HMBS Mutations

**Figure 1:**
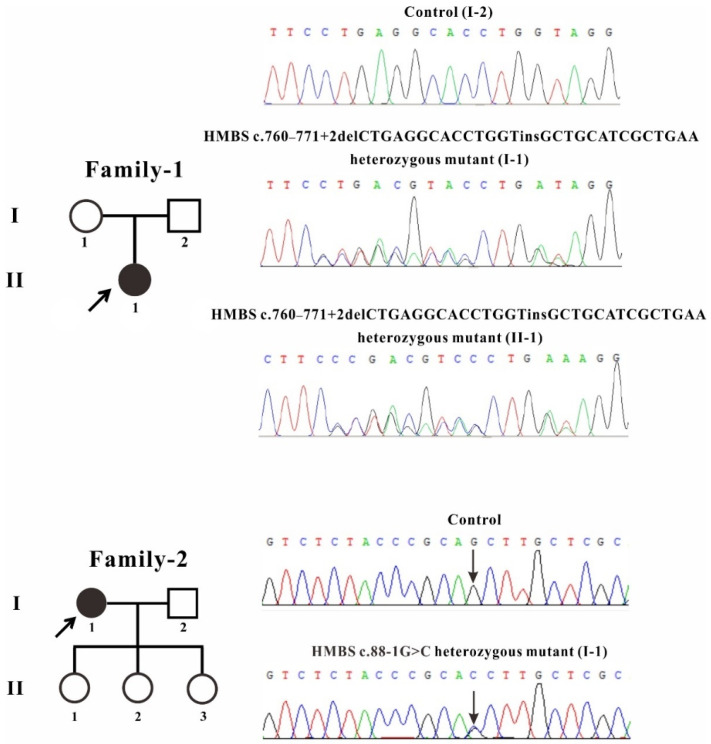
Pedigree and sequencing of patients from two unrelated Chinese families. The patient involved in this study is pointed by an arrow. Sanger sequencing analysis performed on the genomic DNA from indicated patients. The gene variation is shown by a black arrow.

The two AIP patients came from two unrelated Chinese families. After Sanger sequencing, the proband from family 1 inherited a heterozygous *HMBS* mutation (c.760–771+2delCTGAGGCACCTGGTinsGCTGCATCGCTGAA) from his mother (Figure 1). The mutation is located at the end of exon 11 and the head of intron 11 of the *HMBS* gene. The proband presents with intestinal obstruction and confusion during the acute attack. In addition, we confirmed the second novel splicing mutation c.88-1G>C located in the intron 2 of *HMBS* gene in the proband (I-1) of family 2 (Figure 1).

### 2.2. Corroboration of HMBS Mutations as the Determinant of Alternative Splicing in Splicing Assay

**Figure 2:**
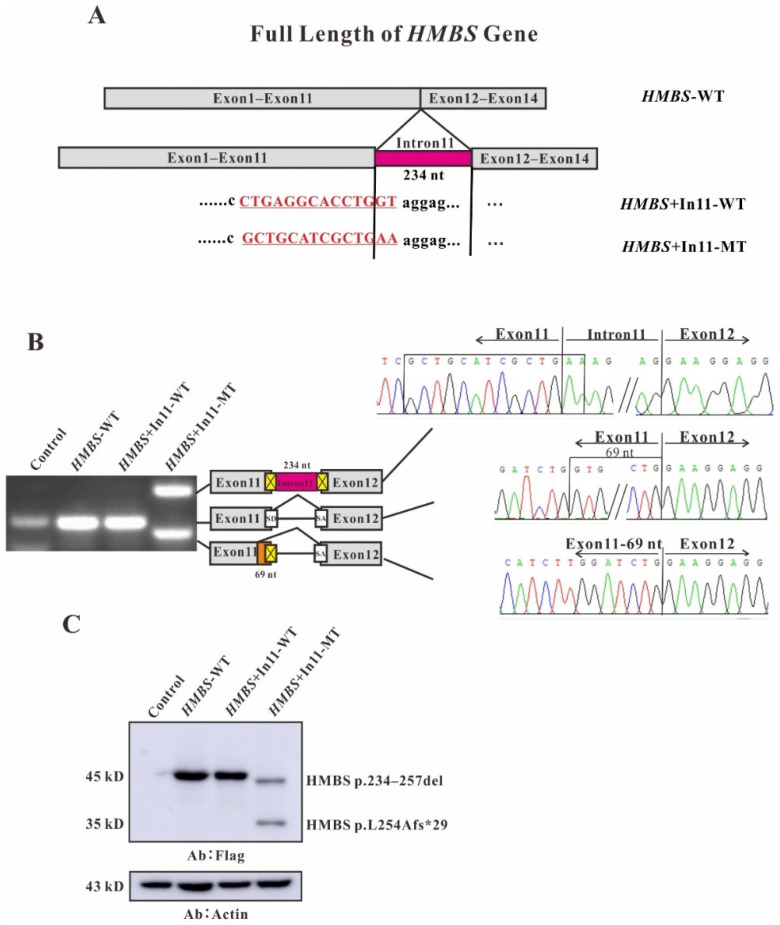
Full length with intron 11 of *HMBS* gene constructs for splicing pattern investigation. (**A**) Schematic representation of the *HMBS* gene with intron 11 construct. The nucleotide sequences of the *HMBS*+In11-WT and *HMBS*+In11-MT are shown below. The changed nucleotide in exon 11 and intro 11 of both wildtype and mutant *HMBS* full gene are labeled by red and underscore. (**B**) Gene splicing assay. *HMBS*-WT, *HMBS*+In11-WT and *HMBS*+In11-MT were transiently transfected into HEK293T cells. After RNA isolation the splicing products were analyzed by RT-PCR. The middle bands represent correct splicing, whereas the higher band represents the *HMBS* inserted 234 nt, the full length of intron 11 and the lower bands represent the *HMBS* missing 69 nt in exon 11. (**C**) Western blot analysis for the expression of *HMBS*-WT, *HMBS*+In11-WT and *HMBS*+In11-MT. Whole cell lysates were separated by SDS-PAGE (12% acrylamide). Flag monoclonal antibodies were used.

**Figure 3:**
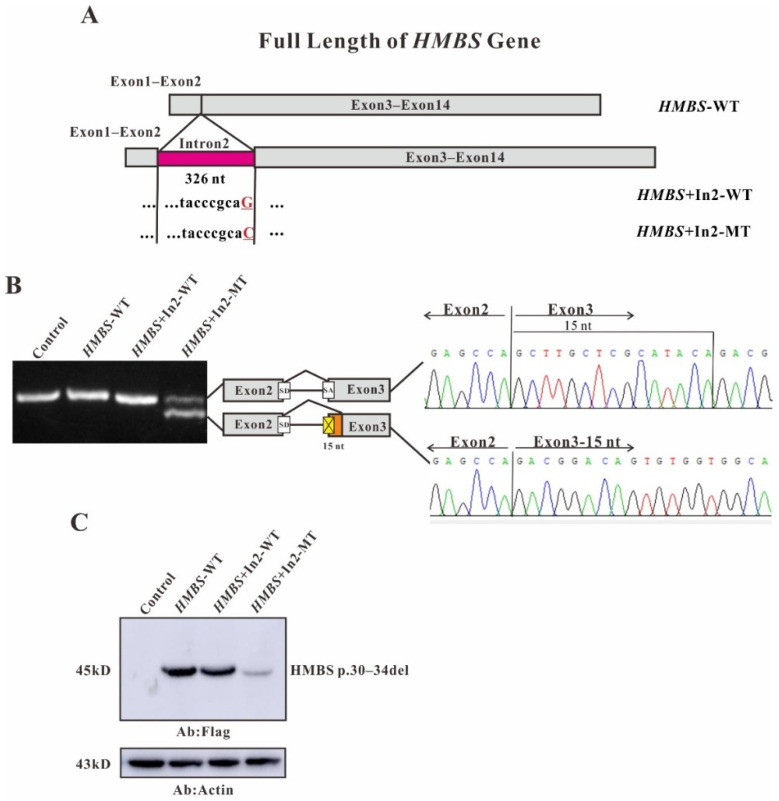
Full length with intron 2 of *HMBS* gene constructs for splicing pattern investigation. (**A**) Schematic representation of the *HMBS* gene with intron 2 construct. The nucleotide sequences of the *HMBS*+In2-WT and *HMBS*+In2-MT are shown below. The changed nucleotide in intro 2 of both wildtype and mutant *HMBS* full gene are labeled by red and underscore. (**B**) Gene splicing assay. *HMBS*-WT, *HMBS*+In2-WT and *HMBS*+In2-MT were transiently transfected into HEK293T cells. After RNA isolation the splicing products were analyzed by RT-PCR. The higher bands represent correct splicing, whereas the lower band represents the *HMBS* missing 15 nt in exon 3. (**C**) Western blot analysis for the expression of *HMBS*-WT, *HMBS*+In2-WT and *HMBS*+In2-MT. Whole cell lysates were separated by SDS–PAGE (12% acrylamide). Flag monoclonal antibodies were used.

Using the Hieff clone one-step rapid cloning kit, four eukaryotic overexpression plasmids of *HMBS*+In11-WT, *HMBS*+In11-MT were constructed, successfully (Figure 2A). After transfection of HEK293T cells, total RNA was isolated and reverse transcription was performed. The RT-PCR products were separated by electrophoresis analysis and identified by Sanger sequencing. In untransfected cells and cells transfected with *HMBS*-WT or *HMBS*+In11-WT, a single band (~256 bp) of the same size was visualized on gel electrophoresis which is consistent with the normal spliced mRNA of the *HMBS* gene. While, the cells transfected with *HMBS*+In11-MT showed two different bands with the molecular weight as 490 and 187 bp on gel electrophoresis. The upper band refers to a product with a 69-nucleotide deletion at the end of exon 11 and the lower band refers to a nucleotide chain retaining the whole intron 11 containing the mutation region (Figure 2B). In order to further study the effect of the mutations on protein expression, Western blot analysis was performed. The protein was extracted from the transfected HEK293T cells and transferred to polyvinylidene fluoride membrane (PVDF membrane) after electrophoresis, and the imprint was detected with Flag antibody. In cells transfected with *HMBS*-WT and *HMBS*+In11-WT, only one band with normal size was observed, while in cells transfected with *HMBS*+In11-MT, two abnormal bands were observed. The upper band is close to the normal size, indicating a translation product from the mRNA with a 69 bp deletion due to abnormal splicing, named as HMBS p.234–257del. Additionally, the lower band is about 35 kD, indicating a translation product from the mRNA retaining the whole intron 11 containing the mutation region due to abnormal splicing. According to the principle of triple codon, translation will stop at the stop codon TGA position in intron 11, named as HMBS p.L254Afs*29 (Figure 2C). Similarly, plasmids of *HMBS*+In2-WT, *HMBS*+In2-MT were constructed (Figure 3A). Both untransfected cells and cells transfected with *HMBS*-WT or *HMBS*+In2-WT showed the bands with the same size (131 bp) indicating the normal splicing of *HMBS*. However, the sequence of the cells transfected with *HMBS*+In2-MT showed a 15-nucleotide deletion at the beginning of exon 3 (Figure 3B). In addition, the band observed from western blot analysis of cells transfected with intron 2 mutant was slightly with lower molecular weight than normal, which due to abnormal splicing resulted in the loss of 15 nucleotides at the head of exon 3, named as HMBS p.30–34del (Figure 3C).

### 2.3. Model Analysis of Abnormal Structure of HMBS Caused by Mutations

**Figure 4:**
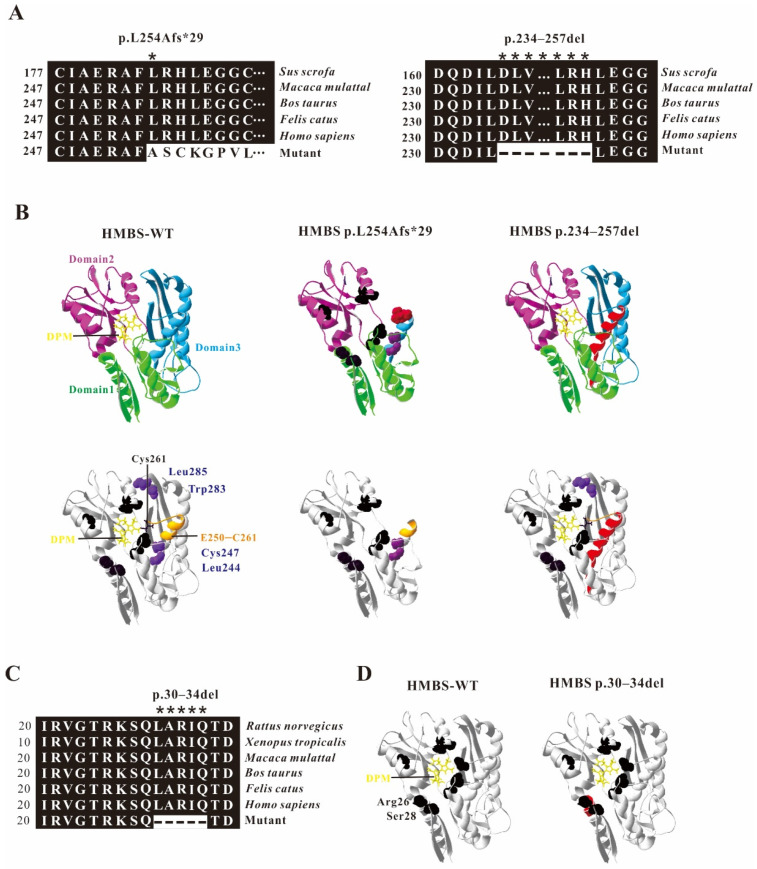
Analysis of HMBS mutations. (**A**) Evolutionary conservation of amino acid residues altered by c.760–771+2delCTGAGGCACCTGGinsGCTGCATCGCTGAA (p.234–257del and p.L254Afs*29) across different species. NCBI accession numbers are *Rattus norvegicus*: NP_037300.2; *Xenopus tropicalis*: NP_001005635.1; *Macaca mulatta*: NP_001253589.1; *Bos taurus*: NP_001039672.1; *Felis catus*: NP_001171279.1; *Homo sapiens*: NP_000181.2. Asterisk (*) means different or missed amino acids. (**B**) Ribbon representation of the HMBS-WT and map of HMBS p.L254Afs*29 and HMBS p.234–257del by homology modeling analysis. HMBS is a monomer enzyme with three domains as pointed out. The active site is shown in black, the mutant or missing amino acids is shown in red; the sliding ring E250–C261 is shown in orange, and the amino acids involved in structural stability are shown in blue. (**C**) Evolutionary conservation of amino acid residues altered by c.88-1G>C (p.30–34) across different species. (**D**) Ribbon representation of the HMBS-WT and map of HMBS p.30–34del by homology modeling analysis. HMBS is a monomer enzyme with three domains as pointed out. The active site is shown in black, the missing amino acids is shown in red.

The crystal structure of human HMBS shows that it is composed of three different α/β domains of similar size, each with about 110 residues. The substrate binding site is located in the cleft between domain 1 and domain 2. Domain 3 mainly plays a role in stabilizing the protein conformation [18,19]. DPM is covalently linked to C261, as a cofactor of HMBS, plays a role in initiating the reaction in the catalysis process, and interacts with multiple amino acids (Arg26, Ser28, Ser96, Asp99, Lys98, Ser147, Arg149, Arg150, Arg173) to form an enzyme activity center. The crystal structure of human HMBS shows that it is composed of three different α/β domains of similar size, and the active site is located between domains 1 and 2 [18,19]. Through amino acid sequence alignment, we found that the *HMBS* c.760–771+2delCTGAGGCACCTGGinsGCTGCATCGCTGAA mutation caused 23 amino acid deletions of HMBS protein (HMBS p.234–257del) and premature termination of HMBS protein (HMBS p.L254Afs*29). The evolutionary conservation analysis of amino acid residues showed that the damaged amino acids are highly evolutionarily conserved in different species (Figure 4A). Through protein structure analysis, on the one hand, these two HMBS-deficient proteins loss different parts of HMBS domain 3, and the defective proteins are easily degraded due to instability; on the other hand, HMBS p.L254Afs*29 lacked C261, which is crucial for the enzyme activity. The amino acid deletion region of HMBS p.234–257del protein is probably closely related to the extension of C261 (Figure 4B). Through amino acid sequence alignment, we found that the HMBS p.234–257del, c.88-1G>C mutation caused 5 amino acid deletions in HMBS protein (HMBS p.30–34del). The evolutionary conservation analysis of amino acid residues showed that the damaged amino acids are highly evolutionarily conserved in different species (Figure 4C). At the same time, the protein structure analysis showed that the missing amino acid is adjacent to the active site region Arg26 and Ser28 (Figure 4D) indicating that the mutation may cause disease. 

### 2.4. Verification of Abnormal HMBS Enzyme Activities Caused by Mutations

**Figure 5:**
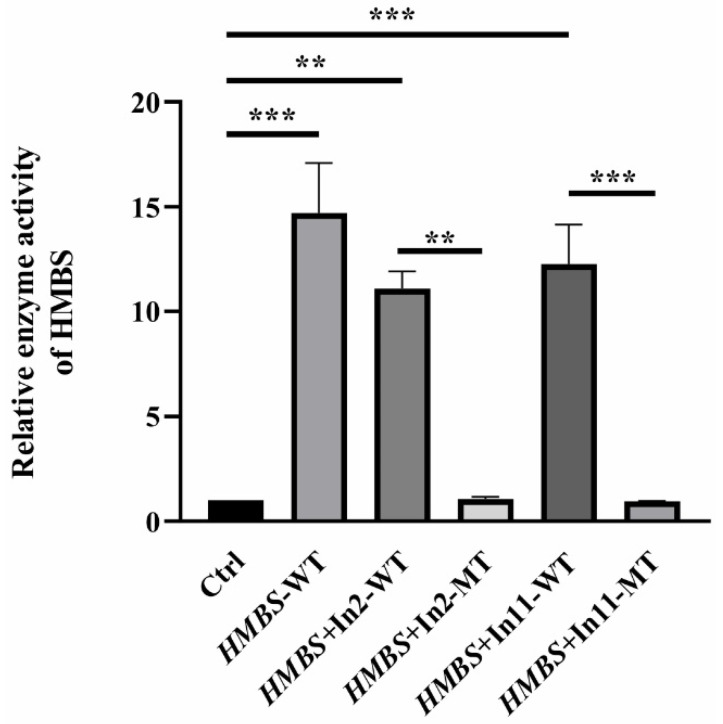
Enzyme activities of the HMBS mutants. The result of enzyme activity assay from HEK293T cells transfected with *HMBS*-WT, *HMBS*+In2-WT, *HMBS*+In2-MT, *HMBS*+In11-WT or *HMBS*+In11-MT plasmids. Data were presented as the mean ± SD from three independent experiments; *** *p* < 0.001; ** *p* < 0.01.

Mutations lead to changes in the structure of the HMBS protein. In order to check whether the mutant proteins contain residual enzyme activity, HEK293T cells were transfected with the same amount of wildtype and mutant *HMBS* plasmids to detect the enzyme activities in vitro. The enzyme activity of untransfected HEK293T cells was set as 1, and the results showed that the enzyme activities of HMBS in cells transfected with *HMBS*-WT, *HMBS*+In11-WT or *HMBS*+In2-WT were greatly enhanced, reaching 10–15 times that of Ctrl. However, the HMBS enzyme activities of cells transfected with *HMBS*+In11-MT or *HMBS*+In11-MT were almost the same as that of untransfected cells, indicating that the two mutant proteins almost lost all enzyme activity (Figure 5).

## 3. Discussion

In this study, we identified two novel mutations affecting consensus-splicing sites of the *HMBS* gene in two unrelated patients diagnosed as AIP. As expected from the low penetrance and the strong phenotypic heterogeneity of the disease, these mutations have been inherited from asymptomatic family members. The c.760–771+2delCTGAGGCACCTGGTinsGCTGCATCGCTGAA mutation is located at the junction of exon 11 and intron 11, and c.88-1G>C is located at the end of intron 2. The coding regions of most human genes are discontinuous and contain multiple exons. After transcription, genes are expressed as pre-mRNAs. Pre-mRNA splicing is a nuclear process in which the intron sequence is excised from the eukaryotic pre-mRNA transcript, and the exons are spliced together to produce a functional gene molecule. For the splicing reaction, the spliceosome must first recognize the splice site at the exon–intron junction, including the splice site donor at the 5′ of the intron and the splice acceptor at the 3′ of the intron [21,22]. Most introns have a 5′ splice site starting with GT and a 3′ splice site ending with AG. Some introns have different splice site consensus sequences and exhibit AT-AC ends or GT-AG ends [23].

For most introns, the border sequences GT and AG are very important for the recognition of the spliceosome [23,24]. In our study, the c.88-1G>C mutation causes the AG at the 3′ end of intron 2 to become AC, the splice acceptor is destroyed, and the spliceosome will identify and enter the next AG, which are at positions 14 and 15 of exon 3. The 15 nucleotides at the head of exon 3 are lost (Figure 3B), resulting in a defective HMBS protein lacking amino acids at positions 30–34 (HMBS p.30–34del) (Figure 3C). For c.760–771+2delCTGAGGCACCTGGTinsGCTGCATCGCTGAA, on the one hand, the mutation causes damage to the 5′ splice donor of intron 11, and the spliceosome misrecognizes the GT located at positions 52–53 of exon 11. The 69 nucleotides at the end of exon 11 are cut (Figure 2B), resulting in a defective HMBS protein lacking amino acids at positions 234–257 (HMBS p.234–257del) (Figure 2C). On the other hand, this complicated mutation may interfere with the recognition of the splice site by the spliceosome, causing a product with all intron 11 retained (Figure 2B). This in-frame insertion produced the premature stop codon TAG at nucleotide 75 downstream of exon 11, resulting in a truncated HMBS protein containing 256 normal amino acids and 29 abnormal amino acids (HMBS p.L254Afs*29) (Figure 2C). However, the normally spliced mRNA was not observed in the cells transfected with *HMBS*+In11-MT (Figure 2B). Considering the different amounts of pre-mRNA transcribed from endogenous *HMBS* and overexpressed *HMBS*-ln11-MT, the band of normal splicing isoform is much weaker to be displayed compared with the abnormal splicing isoforms. Further, cells overexpressing any mutant plasmid show lower HMBS protein expression, although equal amounts of plasmids containing either wildtype or mutant were transfected (Figure 2C and Figure 3C). One explanation could be that the defected protein may be degraded in cell due to the low stability or high cytotoxicity. Another one could be that the transcript of *HMBS*+In11-MT might subject to nonsense-mediated decay (NMD) since the premature termination codons (PTCs) were generated by the mutation [22,25].

Human HMBS consists of three domains. Domain 1 is a non-contiguous domain composed of two regions from residues 1–116 and residues 216–239, residues 117–215 are domain 2, and residues 240–361 constitute domain 3 [19,26]. They are connected by flexible hinge regions (S96, H120, L238). The structural analysis of the two abnormal HMBS proteins (HMBS p.L254Afs*29 HMBS p.234–257del) caused by the c.760–771+2delCTGAGGCACCTGGTinsGCTGCATCGCTGAA mutation showed that the abnormal HMBS protein domain 3 has obvious defects (Figure 4B). HMBS domain 3 is an open-face anti-parallel β-sheet with three chains, one of which is covered by three α-helices. It interacts equally with domains 1 and 2, and plays an important role in maintaining the overall stability of the protein [18,19]. These two defective proteins lose important sites Leu244, Cys247, Leu285, and Trp283 in domain 3 that are involved in maintaining the interaction, so they may be easily degraded and cause under-dose effects [19]. During the catalysis process, HMBS catalyzes the formation of prouroporphyrinogen by combining with DPM cofactor to catalyze porphobilogen. The cofactor covalently binds to the thioether bond of Cys261 and acts as a primer for the reaction [27,28]. The active site cleavage containing DPM cofactor is located at the interface between domains 1 and 2, and forms a catalytically active area by co-acting with Arg26, Ser28, Ser96, Asp99, Lys98, Ser147, Arg149, Arg150, Arg173 [18,19]. It is worth noting that the extension of polypyrrole is a four-time single repeated reaction catalyzed by HMBS using the same catalytic site. The intermediates of the reaction need to be transferred from the active site crack in time, and the slip ring E250–C261 is closely related to it [29]. Therefore, E250–C261 is the key to reaction extension. The amino acid deletion protein HMBS p.30–34del produced by the mutation of c.88-1G>C, its amino acid deletion region is close to the active sites Arg26 and Ser28, which may affect the activity of HMBS protein (Figure 4C). The truncated protein HMBS p.L254Afs*29 produced by the mutation c.760–771+2delCTGAGGCACCTGGTinsGCTGCATCGCTGAA retains the first 253 amino acids of the original HMBS protein. The cofactor DPM cannot bind to it and the catalytic reaction cannot proceed, so the protein is likely to have complete loss of catalytic activity. The defective protein HMBS p.234–257del lacks part of the amino acids in the slip ring E250–C261, which may affect the extension of the reaction and show defects in enzyme activity. To confirm the inference, we measured the HMBS protease activity. Compared with the wildtype *HMBS* transfected cells, the two *HMBS* mutants transfected cells showed almost total loss of HMBS enzyme activity (Figure 5).

## 4. Materials and Methods

### 4.1. Ethical Compliance and Patient Information

This study was approved by the local ethics committee (Ethics Committee of Shanxi University, Ethics number: SXULL2021063, 10 June 2021) and written informed consent was obtained from all participating patients. Both families are from mainland China, and the probands were diagnosed by doctors because of their elevated ALA or PBG levels or clinical symptoms consistent with acute hepatic porphyria [30].

### 4.2. DNA Extraction and Sequencing

DNA was isolated using TIANGEN Blood Genomic DNA Extraction Kit (TIANGEN, Beijing, China Cat No. DP318-03). The DNA was handed over to the Beijing Sinobest Medical Laboratory (Beijing, China) for second generation sequencing, specifically: extracting genomic DNA from the subject’s peripheral blood, constructing a genomic library, and then capturing the penetrance of genes related to genetic diseases through probe hybridization and the adjacent intron region (50 bp), and enriching it. The enriched target gene fragments were sequenced by the next-generation high-throughput sequencer (Illumina, California, CA, USA). The average sequencing depth of the samples is 177.37×. According to the results of second-generation sequencing, the upstream and downstream primers were designed near the mutation site. The sequences of the family1 mutation site verification primers are Fw1: CCTTGGGCGTGGAAGTGC; RV1: TGGGCAGGGACATGGATGG; the family2 mutation site verification primers are Fw2: ATGAGAGTGATTCGCGTGGGTA; Rv2: TCCCCTGTGGTGGACATAGC. Sanger sequencing was performed after PCR amplification. The primer synthesis and sequencing were completed by Sangon Biotech (Shanghai, China).

### 4.3. Construction of In Vitro Expression Vector

The eukaryotic expression vector pcDNA3.1+N-DYK-P2A containing the CDS region of the *HMBS* gene (NM_000190.4) was purchased from Nanjing GenScript Biotechnology Co., Ltd (Nanjing, China). Intron 2 and intron 11 were obtained from the genome by PCR, and a homologous recombination kit was used to construct wildtype and mutant plasmids carrying intron 2 and intron 11, respectively.

### 4.4. Transient Transfection

HEK293T cells were preserved in our laboratory. The cells were plated in a cell culture dish in advance, and when confluence was up to 70–80%, linear polyethyleneimine (PEI; PolyScience, Niles City, IL, USA, Cat.No. 23966-2) was used as a transfection reagent for transient transfection. The transfection time was about 24–48 h.

### 4.5. RT-PCR, PCR and Sequencing

In order to evaluate the effect of mutation sites on mRNA splicing, HEK293T cells with a confluence of 90% were collected, RNA was extracted by Trizol method and reverse transcription was performed. The PCR primer pairs used for *HMBS* are Fw1: CCTTGGGCGTGGAAGTGC; Rv1:TGGGCAGGGACATGGATGG; Fw2: ATGAGAGTGATTCGCGTGGGTA; Rv2:TCCCCTGTGGTGGACATAGC and the PCR products were sent for sequencing. The primer synthesis and sequencing were completed by Sangon Biotech (Shanghai, China).

### 4.6. Western Blotting

HEK293T cells were collected with 95% confluence after transfection, washed twice with cold PBS, resuspended in lysis buffer, lysed on ice for 30 min, sonicated for 3 s. The centrifuged supernatant was taken and resuspended in 5×SDS sample buffer and heated to 98 °C for 5–10 min. A 12% SDS separation gel was used for 120 V constant pressure electrophoresis, and the protein was transferred to the PVDF membrane by a wet blot transfer method for 2 h under a constant current of 0.3 A. The primary antibody (Flag: Proteintech, Wuhan, China, Cat No: 20543-I-AP; Actin: Proteintech, Cat No: 60008-I-Ig) and secondary antibody (Proteintech Cat No: SA00001-2 and Cat No: SA00001-1) were incubated, respectively, and the signal detected by a chemiluminescence system (GE, Hongkong, China, Cat. No. RPN2232).

### 4.7. The Evolutionary Conservation Analysis of Amino Acid Residues and the Structure Prediction of Mutant Proteins

The evolutionary conservation of amino acid residue changes was analyzed by comparison between different species. The homology modeling program Swiss-Model (http://swissmodel.expasy.org, accessed on 11 July 2021) was used to develop a suitable model to simulate the effect of the mutation region. These structures are displayed by the PDB viewer software (SPDBV_4.10_PC).

### 4.8. Determination of HMBS Enzyme Activity

HEK293T cells were cultured and transfected with plasmids containing wildtype or mutant *HMBS* of the same quality, respectively. After 24 h of transfection, the cells were collected by centrifugation at 1500× *g* rpm in a benchtop centrifuge. Every 2 million cells were resuspended in 60 μL lysis buffer, placed on ice for 30 min, and then sonicated. They were centrifuged at 12,000× *g* rpm at 4 °C for 10 min, and 50 μL of the lysate supernatant was collected. As mentioned earlier, the enzyme activity is determined by measuring the conversion of PBG to uroporphyrin [31,32]. In total, 50 microliters of lysate supernatant was mixed with 900 microliters of 1 M Tris-HCl (pH = 8.1), pre-incubated at 37 °C for 3 min, and then incubated with 250 μL of 0.1 mM PBG substrate for 60 min at 37 °C in the dark. The reaction was terminated with 175 mL of cold 40% trichloroacetic acid. After 30 min of exposure to sunlight, absorbance was measured at 405 nm. At the same time, the protein concentration was determined by the BCA method. An appropriate standard was used to express HMBS activity in pmol uroporphyrin/mg protein/h. The measurement was performed three times, and the results were described as average values.

## 5. Conclusions

In summary, we reported and verified two novel *HMBS* pathogenic splicing mutations c.88-1G>C and c.760–771+2delCTGAGGCACCTGGTinsGCTGCATCGCTGAA predispose to AIP. Our research has enriched the AIP mutation database, promoted the molecular diagnosis of AIP family members, and further expanded the molecular heterogeneity of this acute liver porphyria.

## Data Availability

All primary data are available upon reasonable request.
